# A lateral flow microarray immunoassay to detect USUV NS1-specific antibodies in tissues of deceased blackbirds in the Netherlands

**DOI:** 10.1016/j.virusres.2026.199789

**Published:** 2026-08-16

**Authors:** Bijan Godarzi, Cora M. Holicki, Felicity D. Chandler, Anne van der Linden, Reina S. Sikkema, Edwin J.A. Veldhuizen, Aart van Amerongen, Andrea Gröne

**Affiliations:** a Division of Pathology, Department of Biomolecular Health Sciences, Utrecht University, Utrecht, the Netherlands, Yalelaan 1, 3584, CL, Utrecht, the Netherlands; b BioSensing & Diagnostics, Wageningen University and Research, Wageningen, the Netherlands, Bornse Weilanden 9, 6708, WG, Wageningen, the Netherlands; c Department of Viroscience, Erasmus MC, Rotterdam, the Netherlands, Wytemaweg 80, 3015, CN, Rotterdam, the Netherlands; d Division of Immunology & Infectious Diseases, Department of Biomolecular Health Sciences, Utrecht University, Utrecht, the Netherlands, Yalelaan 1, 3584, CL, Utrecht, the Netherlands

**Keywords:** Arboviruses, Avian, Usutu virus, One health, Lateral flow microarray immunoassay (LMIA), Serology

## Abstract

Usutu virus (USUV) is an arbovirus that circulates between mosquitoes and birds with occasional spillover events into non-avian species. USUV continues to circulate in the Netherlands, particularly in Eurasian blackbirds (*Turdus merula*), although other USUV-susceptible avian species have also been found. Current serosurveillance systems make use of serological methods that can detect anti-USUV antibodies in sera collected from live birds. However, they require a dedicated laboratory and rarely include serological analysis of deceased avian specimens. As a proof of concept, this study evaluates the use of a previously developed lateral flow microarray immunoassay (LMIA) to detect USUV non-structural 1 (NS1)-specific antibodies in blood clots and lung and liver tissue extracts collected from blackbird carcasses found in the Netherlands. The likelihood of detection of USUV NS1 antibodies in USUV RT-PCR-positive blackbirds was found to trend higher in lung tissue extracts. Additionally, the LMIA exhibited only minimal cross-reactivity between USUV NS1-specific antibodies and USUV NS1 and West Nile virus (WNV) NS1 capture antigens on the LMIA. This initial study demonstrates the feasibility of the LMIA to specifically detect USUV NS1 antibodies in lung tissue extracts of deceased blackbirds. In future, a serological tool such as the one presented here, would allow for the rapid on-site analysis of both live and deceased blackbird specimens found in the field.

## Highlights

• Novel lateral flow microarray used to detect USUV antibodies in post-mortem tissues.
• USUV NS1 antibodies were detected in lung, liver, and blood clot-derived samples.
• Lung samples showed higher detection rates than matched liver samples.
• Performance was validated against protein microarray and FRNT_90_.

## 1 Introduction

Arboviruses are viruses that are transmitted by arthropods such as ticks and mosquitoes and are considered emerging pathogens due to their geographical distribution and incidence rates continuing to increase. Usutu virus (USUV) is an arbovirus in the *Flaviviridae* family that has been shown to circulate in an enzootic cycle whereby birds act as amplifying hosts and *Culex* mosquitoes act as vectors. Via mosquito bites, USUV can occasionally spill over into horses and humans which act as dead-end hosts (Nikolay, 2015).

USUV was first detected in the Netherlands in 2016, primarily in Eurasian blackbirds (*Turdus merula*), and was subsequently detected in Dutch blood donors (Rijks et al., 2016; Zaaijer et al., 2019). Consistent with recurring seasonal transmission in multiple avian species, major outbreaks in birds were reported until 2018 and renewed circulation in 2022. Eurasian blackbirds are consistently associated with both high infection prevalence and mortality, making them a key sentinel species for surveillance (Münger et al., 2025). Furthermore, other USUV-susceptible avian species found in the Netherlands, including but not limited to, song thrushes (*Turdus philomelos*), Eurasian jays (*Garrulus glandarius*), house sparrows (*Passer domesticus*), and blue tits (*Cyanistes caeruleus*), have also been reported (Münger et al., 2025). West Nile virus (WNV) was first detected in the Netherlands in 2020 in a common white throat (*Curruca communis)* followed by detections in mosquitoes and humans with subsequent serological evidence suggesting ongoing but early-stage circulation (Münger et al., 2025; Sikkema et al., 2020; Vlaskamp et al., 2020). Currently, a surveillance system is in place for both flaviviruses that combines molecular and serological surveillance of live birds with an additional molecular screening of deceased birds.

While molecular methods such as RT-PCR enable sensitive detection of viral RNA in tissues from deceased birds, they rely on the presence of viral material at the time of sampling. In contrast, serological methods detect host antibodies, which can persist beyond viral clearance and thereby extend the diagnostic window. At present, serological surveillance is primarily performed in live birds using serum or plasma samples, whereas deceased birds are typically assessed only by molecular methods. As a result, past infections in carcasses may remain undetected. Extending serological testing to post-mortem samples could therefore complement existing surveillance approaches, particularly in scenarios such as mass mortality events in the Netherlands where large numbers of carcasses are available through non-targeted (passive) surveillance networks (Rijks et al., 2016). Additionally, carcasses collected through non-targeted (passive) surveillance networks do not require prior ethical approval and administrative procedures (Streng et al., 2023).

However, the application of serology to deceased birds presents practical challenges. Standard assays such as enzyme-linked immunosorbent assays (ELISA) and neutralization tests (NT) typically require serum or plasma, which is difficult to obtain from carcasses due to blood clotting (Campbell and Dein, 1984). Although the detection of flavivirus-specific antibodies in tissue-derived samples has been reported (Meister et al., 2008), the feasibility and performance of such approaches remain insufficiently explored. Exploring whether antibodies can be reliably detected in post-mortem tissues is therefore a necessary first step before considering their use in field surveillance.

Lateral flow assays (LFA) are an established, nearly equipment-free method that allows for the rapid and on-site analysis of a given sample. To enable the detection of multiple analytes, the test line in LFAs is replaced by a microarray of small test spots (van Amerongen and Koets, 2025) (Supplementary Figure S1). In a previous study, a species-independent WNV and USUV non-structural 1 protein (NS1) lateral flow microarray immunoassay (LMIA) was developed and demonstrated the ability to detect and differentiate between WNV NS1 or USUV NS1 antibodies in the sera of humans, horses, blackbirds, and jackdaws (Godarzi et al., 2024). In the present study, whether this previously developed LMIA can detect USUV NS1-specific antibodies in post-mortem samples from field-collected blackbirds was evaluated. As a proof of concept, blood clots and tissue extracts (lung and liver) were tested to assess the feasibility of antibody detection in these matrices. LMIA performance was benchmarked against NS1 protein microarray (PMA) and 90% focus reduction neutralization test (FRNT_90_) reference methods to provide initial insight into assay performance across different serological platforms.

Although the current workflow is laboratory-based, demonstrating antibody detectability in post-mortem tissues is a critical first step toward the potential development of simplified, field-adapted sampling and testing strategies. Such approaches could complement existing surveillance systems by enabling broader use of carcass-derived samples for serological analysis.

## 2 Materials and methods

### 2.1 Blackbird tissue sample selection

As part of ongoing surveillance efforts in the Netherlands, deceased blackbirds around the country are reported and submitted to the Dutch Wildlife Health Centre (DWHC) where they are examined and, if possible, necropsied to determine the cause of death. During necropsy, a variety of biological samples are taken and stored for later analysis. These samples include, but are not limited to, tissue samples of the brain, lung, and liver and occasionally blood clots from near the heart (Supplementary Figure S2). As part of the post-mortem analysis for the surveillance of arboviruses in deceased birds, brain tissue samples are sent to the Erasmus Medical Centre (EMC) where they are tested for the presence of USUV via real-time PCR (RT-PCR) as described (Atama et al., 2022) (Supplementary Figure S3).

In collaboration with the DWHC, RT-PCR results of collected brain tissue samples from deceased blackbirds found in the Netherlands between 2020 and 2022 were shared. Data on the seropositivity of deceased blackbirds or their tissue extracts are not available. Therefore, to maximise the chance of selecting specimens that could have developed an immune response to infection, Ct values of brain tissue samples were used as a second-best option during the sample selection process. Tissue samples from deceased blackbirds with confirmed USUV RNA presence in the brain were selected for sample preparation and serological analysis using the LMIA. Initially, aliquots of all the lung and liver tissues and blood clots corresponding to all the USUV RT-PCR-positive confirmed blackbirds were requested. However, only a small subset (n = 23) of the original stocks was still available for selection (Supplementary Figure S3).

Similarly, blackbirds whose brain tissue USUV RT-PCR results came back negative (n = 5) were selected as negative controls (Supplementary Figure S3). All selected tissue samples were then aliquoted at the DWHC and sent to the EMC for preparation and analysis using the LMIA, PMA, and FRNT_90_. A condensed overview of all tissue extracts collected from deceased blackbirds used in this study are found in Table 1.

**Table 1:** Overview of blackbird tissue extracts used in this study.

Tissue	Brain USUV RT-PCR	# extracts	Confirmatory test	Origin
Blood clots	Positive	9*	USUV and WNV FRNT_90_	NL
	Negative	2	USUV and WNV FRNT_90_	NL
Lung	Positive	18	USUV and WNV FRNT_90_	NL
	Negative	5	USUV and WNV FRNT_90_	NL
Liver	Positive	18	USUV and WNV FRNT_90_	NL
	Negative	5	USUV and WNV FRNT_90_	NL
Total		57		

⁎ One excluded from analysis. Abbreviations: FRNT_90_ = 90% focus reduction neutralization test; NL = the Netherlands.

### 2.2 Production of USUV NS1 and WNV NS1 LMIA

The USUV NS1 and WNV NS1 LMIA used in this study was prepared as previously described including antigen production, conjugation of antigen to carbon nanoparticles (CNP), and preparation of optimized LMIAs (Godarzi et al., 2024). Briefly, the optimized LMIAs used in this study were comprised of a sample/conjugate pad containing USUV NS1, WNV NS1, and BSA-DNP detection conjugates each at 350 µg/mL; a nitrocellulose membrane spotted with duplicate USUV NS1 and WNV NS1 spots at 3000 µg/mL (PBS, pH 8.8), duplicate control spots (anti-DNP KLH) at 2000 µg/mL, and four guide spots (polymerInk VP240 blue non-soluble dye); and an absorbent pad (Supplementary Figure S1).

### 2.3 Preparation of blood clot and tissue samples

Selected blood clot, lung and liver tissue samples were prepared as previously described (Sikkema et al., 2022). Briefly, selected tissue samples were thawed on ice, tissue fragments (0.1 – 3 cm^3^) placed into 15 mL conical tubes with 2 mL PBS, and left on ice for 20 min. Next, samples were rotated at 4 °C for 20 min and centrifuged at 4 °C for 15 min at 1000 x g. The tissue supernatant was then collected for analysis (Supplementary Figure S1).

Prior to testing on LMIA, PMA, and FRNT_90_ and to increase the sensitivity of the LMIA, the collected supernatant was concentrated approximately 20-fold using Amicon® Ultra-0.5 Centrifugal Filter Devices (Merck, Darmstadt, Germany). Although this concentration step reduces user-friendliness in a field setting, it was included as part of this proof-of-concept study (Supplementary Figure S1).

### 2.4 Detection of antibodies in tissue extracts using LMIA

The detection of antibodies using the USUV NS1 and WNV NS1 LMIA was done as previously described with some modifications (Godarzi et al., 2024). Briefly, tissue extracts were diluted 1:5 in assay running buffer (borate buffer, 100 mM, 1% (*w/v*) skim milk protein, 0.05% (*v/v*) Tween20, pH 8.8) and 100 µL pipetted onto the sample/conjugate pad of the LMIA placed in a plastic cassette. A sciREADER LF1 real-time video reader (Scienion, Berlin, Germany) measured the spot intensity of the test and control spots after 20 min.

LMIA results were considered qualitatively LMIA-reactive if spot development was observable by eye after 20 min, whereas results were considered quantitatively LMIA-reactive if spot intensity (AU), as measured by the LF1 reader after 20 min, was greater than 0. Similarly, LMIA results were considered qualitatively LMIA-non-reactive if spot development was not observable by eye and quantitatively LMIA-non-reactive if spot intensity (AU) was 0. Categorical quantitative results (i.e. LMIA-reactive or LMIA-non-reactive) were used for comparisons between tissue types.

### 2.5 USUV NS1 and WNV NS1 protein microarray (PMA)

PMA was performed as previously described with modifications (Münger et al., 2025). 64 nitrocellulose pad glass slides (Sartorius, Göttingen, Germany) were spotted with USUV NS1 (The Native Antigen Company) and WNV NS1 (Sino Biological) in duplicate. For IgY antibody detection, slides were incubated for 1 h at 37 °C with the two-fold diluted blackbird tissue extracts ranging from 1:20 to 1:2560. Subsequently, slides were incubated for 1 h at 37 °C with Alexafluor-647 conjugated goat anti-duck IgY 647 (Jackson Immunoresearch Laboratories, Inc., West Grove, USA).

PMA results were classified as PMA-non-reactive, USUV PMA-reactive, or WNV PMA-reactive based on virus-specific cut-off titres. Extracts with both USUV NS1 IgY and WNV NS1 IgY titres below their respective thresholds (USUV NS1 IgY < 20; WNV NS1 IgY < 20) were considered PMA-non-reactive. USUV PMA-reactive extracts had an USUV NS1 IgY titre ≥ 20 while WNV PMA-reactive extracts had a WNV NS1 IgY titre ≥ 20.

### 2.6 90% focus reduction neutralization test (FRNT_90_)

FRNT_90_ was performed using WNV lineage 2 (B956, NCPV Porton Down #638, 2010) and USUV Africa-3 (*Turdus merula* NL isolate, 2016, EVAg Ref-SKU: 011V-02,153) as previously described with modifications (Münger et al., 2025). While a standard FRNT_90_ protocol tests serum that has been separated from a whole blood sample, the present study tested tissue extracts instead, as serum samples from deceased blackbirds were not available for testing.

FRNT_90_ results were classified as seronegative, USUV-seropositive, WNV-seropositive, or flavivirus-seropositive based on virus-specific cut-off titres and relative titre differences. Extracts with both USUV and WNV titres below their respective thresholds (USUV < 160; WNV < 80) were considered seronegative. USUV-seropositive extracts had an USUV titre ≥ 160 and at least a fourfold higher than the WNV titre; WNV-seropositive extracts had a WNV titre ≥ 80 and at least a fourfold higher than the USUV titre. Extracts above cut-off but with less than a fourfold difference were classified as flavivirus-seropositive.

### 2.7 Data analysis

Data analysis for optimized LMIAs was performed as previously described with some modifications (Godarzi et al., 2024). The sciREADER LF1 spot intensity measurements are expressed in arbitrary units (AU) and are background corrected by the reader’s software.

All subsequent data analysis was done using GraphPad Prism (version 9.3.1) (GraphPad Software, 2021) or R software (version 4.2.3) (R Core Team, 2023) and packages: ggplot2 (Wickham, 2016), dplyr (Hadley Wickham, 2023), and tidyverse (Hadley Wickham et al., 2019) as previously described with some modifications (Godarzi et al., 2024). For each LMIA test, individual test spot intensities were corrected with respect to the average of internal control spot intensities. For each sample type (blood clots, lung or liver tissue), a Wilcoxon signed-rank test was used to determine the significance of difference between mean USUV NS1 and WNV NS1 spot intensities (*p* < 0.05).

McNemar’s test was used to evaluate whether differences in categorical quantitative USUV NS1 LMIA results between paired lung and liver tissue extracts were statistically significant (*p* < 0.05). Fisher’s exact test was used to assess the significance of associations between USUV NS1 LMIA and FRNT_90_ results, and between USUV NS1 LMIA and USUV NS1 IgY PMA results (*p* < 0.05).

## 3 Results

### 3.1 Overview of deceased blackbird samples tested

In total, 23 USUV RT-PCR-positive blackbirds had tissue samples available for analysis. Of those 23, 4 had a blood clot, a lung tissue, and a liver tissue sample available; 14 had only a lung and a liver tissue available; and 5 had only a blood clot available. Thus, a total of 9 blood clots, 18 lung tissue, and 18 liver tissue samples from USUV RT-PCR-positive blackbirds were available for analysis. However, as described later, the spot intensity (AU) of one of the blood clot samples exceeded the upper interquartile range (IQR) boundary by more than six times, and was considered an outlier and excluded from analysis. Thus, a total of 22 USUV RT-PCR-positive blackbirds were included in data analysis.

The selected USUV RT-PCR-positive blackbirds were found deceased in the months of August and September 2020, 2021, and 2022 with the majority found in 2022 (12/22). During 2022, most of the deceased USUV RT-PCR-positive blackbirds (8/12) were found in the central and southern provinces of Utrecht, Gelderland, and North Brabant. These spatiotemporal data were not unexpected as USUV outbreaks in the Netherlands are known to be seasonal with most USUV RT-PCR-positive deceased blackbirds being reported in August and September and a resurgence, especially in the central and southern provinces, being reported in 2022 (Münger et al., 2025).

Of the 5 USUV RT-PCR-negative blackbirds that were analysed, 2 had a blood clot, a lung tissue, and a liver tissue available while 3 had only a lung and a liver tissue available.

### 3.2 Comparison of USUV NS1 LMIA detection by RT-PCR status and tissue type

To determine how USUV NS1 LMIA results compared to USUV RT-PCR status and which tissue types showed the highest likelihood of detecting USUV NS1 antibodies, LMIA results were stratified based on USUV RT-PCR status and tissue type (Table 2). Comparison across tissue types is based on categorical quantitative results using the reader as referenced in the methods 2.4. Amongst all USUV RT-PCR-positive extracts tested, 9 of 18 lung (50%), 4 of 18 liver (22%), and 3 of 8 blood clot extracts (38%) were also USUV LMIA-reactive. Across all 12 USUV RT-PCR-negative extracts tested, only 1 lung extract was USUV LMIA-reactive. USUV LMIA results were heterogeneous across tissue types, with several blackbirds being LMIA-reactive in a subset of the available samples (Supplementary Table S4). Among the USUV RT-PCR-negative blackbirds (n = 5), all but one blackbird was LMIA-non-reactive across the available tissues; this one individual was USUV LMIA-reactive in the lung only (Supplementary Table S4).

**Table 2:** A comparison of USUV LMIA results stratified by USUV RT-PCR status and tissue type.

		USUV RT-PCR					
		Blood Clot		Lung		Liver	
		Positive	Negative	Positive	Negative	Positive	Negative
USUV LMIA	Reactive	3	0	9	1	4	0
	Non-reactive	5	2	9	4	14	5

When comparing lung to liver USUV LMIA results, irrespective of RT-PCR status, (n = 23 blackbirds), 9 lung samples (39%) and 4 liver samples (17%) were USUV LMIA-reactive. A paired analysis of lung and liver extracts from the same blackbirds indicated more cases that were USUV LMIA-reactive in lung but LMIA-non-reactive in liver (7/23) compared with the reverse (1/23), although this difference showed a non-significant trend (McNemar’s test, *p* = 0.07). The paired odds ratio was 7.0 (95% CI: 0.86–57.1), suggesting that lung tissue may be more likely than liver to yield USUV LMIA-reactive results, although the confidence interval was very wide, likely due to the small number of discordant pairs.

When comparing lung to blood clots USUV LMIA results, irrespective of RT-PCR status (n = 6 blackbirds), 4 blackbirds were LMIA-non-reactive in both tissues, one was USUV LMIA-reactive in both tissues, and one was USUV LMIA-reactive in blood clot only (Supplementary Table S4). Due to the small sample size, the odds ratio analysis and McNemar’s test could not be performed.

Taken together, these results suggest a trend towards a higher likelihood of USUV NS1 antibody detection in lung compared to liver. However, given the lack of statistical significance and wide confidence intervals, further studies with larger sample sizes are required to confirm this finding.

### 3.3 Minimal cross-reactivity observed between USUV NS1-specific antibodies and WNV NS1 capture antigen

To determine if significant differences could be observed between USUV NS1 and WNV NS1 spot intensities (AU)**,** all 17 USUV LMIA-reactive tissue extracts were categorized with respect to tissue type (blood clot, liver, or lung) and Wilcoxon signed-rank tests were performed to determine the levels of significance between USUV NS1 and WNV NS1 LMIA spot intensities (AU).

**Fig. 1:**
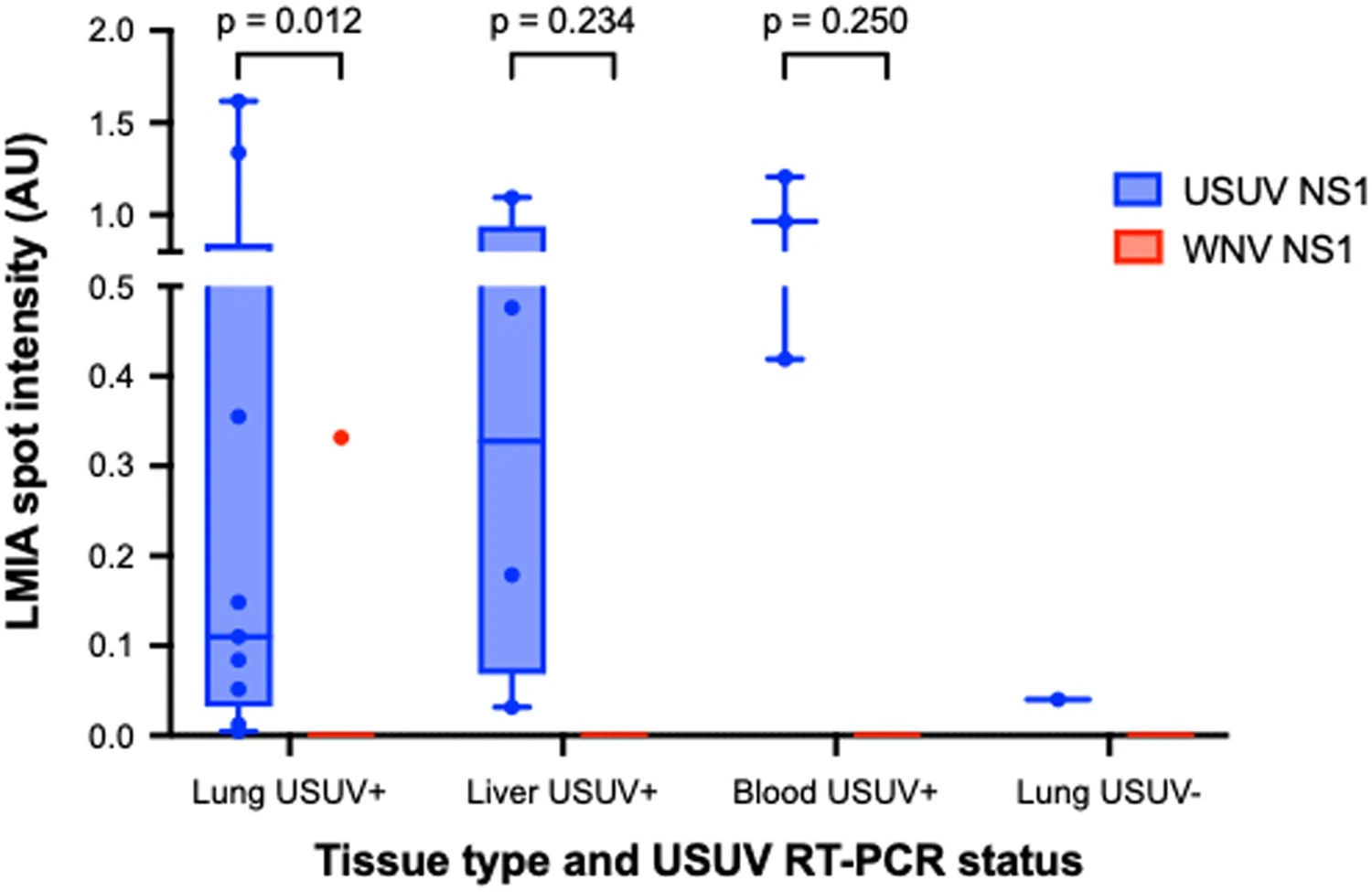
Statistically significant differences observed between USUV NS1 and WNV NS1 spot intensities in lung tissue of USUV RT-PCR-positive blackbirds. The box plots depict USUV NS1 and WNV NS1 LMIA spot intensities (AU) as measured by the LF1 reader after 20 min for Lung USUV+ (n = 9), Liver USUV+ (n = 4), Blood clot USUV+ (n = 3), and Lung USUV- (n = 1). For each individual extract tested, spot intensities are shown as dots. The median values of USUV NS1 for Lung USUV+, Liver USUV+, Blood USUV+, and Lung USUV- are 0.11, 0.32, 0.97, and 0.04, respectively. The median values of WNV NS1 for all categories are 0. Comparison of USUV NS1 and WNV NS1 spot intensities for each category (Wilcoxon signed-rank test, *p* < 0.05).

Across all 17 USUV LMIA-reactive tissue extracts, the median spot intensity for a homologous binding (i.e. USUV antibodies binding USUV NS1 antigens) event was higher than it was for a heterologous one (i.e. USUV antibodies binding WNV NS1 antigens) (Fig. 1). However, a statistically significant difference between USUV NS1 and WNV NS1 spot intensity was only observed within the USUV RT-PCR-positive lung tissue extract sub-category (Wilcoxon signed-rank test; *p* = 0.004). Although the differences between USUV NS1 and WNV NS1 median spot intensities were greater in the USUV RT-PCR-positive blood clot and liver sub-categories than in the lung sub-category, these differences were not statistically significant (Wilcoxon signed-rank test; *p* = 0.250 and 0.125, respectively). The absence of significance in the blood clot and liver datasets likely reflects the smaller sample sizes, which limited the statistical power to detect differences.

Taken together, the LMIA exhibited minimal cross-reactivity between USUV NS1-specific antibodies found in lung tissue extracts of USUV RT-PCR-positive blackbirds and WNV NS1 LMIA capture antigens. However, a larger sample set that also includes samples from known WNV-seropositive blackbirds would further strengthen and expand this conclusion to assess minimal cross-reactivity between USUV NS1 capture antigens and WNV NS1-specific antibodies.

### 3.4 USUV NS1 LMIA results can be treated as categorical

**Fig. 2:**
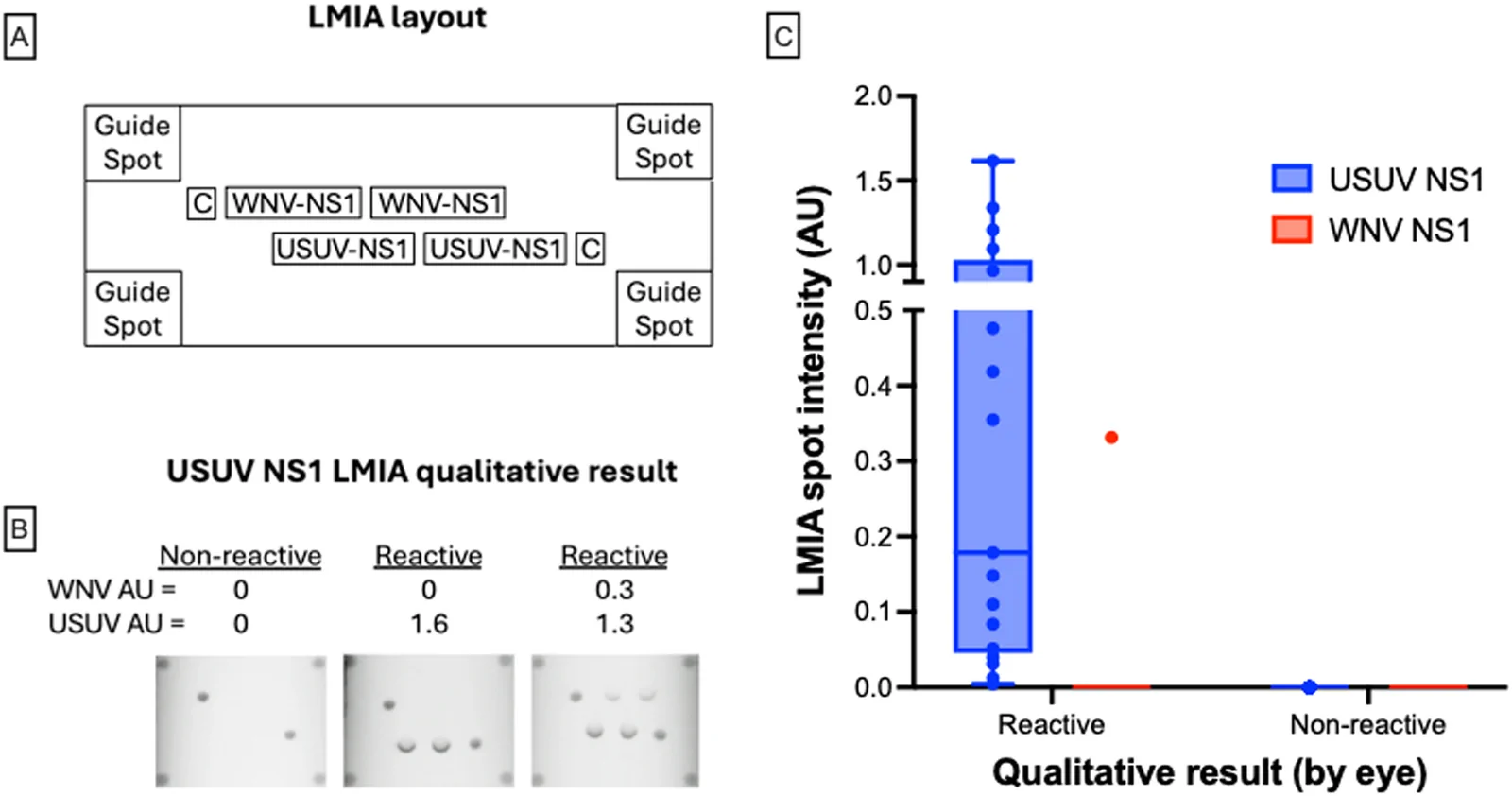
USUV LMIA results can be treated as categorical. A. Schematic layout of a single LMIA strip (C: Control). The LF1 reader uses guide spots to find the grid, and these guide spots play no role in analysis. B. Examples of qualitative results after 20 min as captured by the LF1 reader. The corrected, mean USUV NS1 and WNV NS1 spot intensities (AU) are above each corresponding image. C. Boxplot comparing qualitative (x-axis, LMIA-reactive or LMIA-non-reactive by eye) and quantitative results (y-axis, corresponding spot intensity as measured by LF1 reader) after 20 min. For all qualitatively LMIA-reactive (n = 17) and LMIA-non-reactive (n = 39) results, the spot intensities (AU) are greater than 0 and equal to 0, respectively.

To evaluate if USUV LMIA outcomes can be treated as categorical without requiring a defined signal cut-off, distributions of quantitative LMIA spot intensities were compared between qualitatively LMIA-reactive and LMIA-non-reactive results. From the 22 USUV RT-PCR-positive blackbirds, results from a total of 44 tissue extracts were compared. Of these, 16 tested both qualitatively and quantitatively LMIA-reactive and 28 tested both qualitatively and quantitatively LMIA-non-reactive. From the 5 USUV RT-PCR-negative blackbirds, results from a total of 12 tissue extracts were compared. Of these, 1 tested both qualitatively and quantitatively LMIA-reactive and 11 tested both qualitatively and quantitatively LMIA-non-reactive (Fig. 2b).

Across all LMIA-reactive tissue extracts (n = 17), the distribution of spot intensity (AU) ranged from a minimum of 0.01 to a maximum of 1.6169, with a median of 0.3316. The IQR spanned from 0.0841 to 0.9657, indicating the middle 50% of values. Of these 17 LMIA-reactive samples, one tested LMIA-reactive for both USUV NS1 and WNV NS1 antibodies (Fig. 2b). Reproducibility assessed across duplicate capture spots was generally high for samples yielding strong signals, but variation increased for weakly positive samples. In these weakly positive samples, the spot development remained visually detectable, although the corresponding quantitative values showed higher coefficients of variation (Supplementary Table S4).

Although the use of the LF1 reader allows for a more objective readout and data sharing, these findings indicate that qualitative visual interpretation closely matched the quantitative reader output in this dataset, supporting the use of LMIA as a categorical screening assay under the conditions tested.

### 3.5 Evaluation of LMIA diagnostic performance compared to FRNT_90_ and PMA

The diagnostic performance of the LMIA was also compared to two other serological methods. The LMIA results of 55 tissue extracts from 26 blackbirds were compared to “gold standard” USUV and WNV FRNT_90_ results. One lung tissue and one blood clot extract did not have enough material to perform FRNT_90_ and was thus excluded. Additionally, the LMIA results of 21 tissue extracts from 17 RT-PCR-positive blackbirds were compared to USUV NS1 and WNV NS1 IgY PMA results. The remaining tissue extracts did not have enough material to perform PMA.

Based on the classifications mentioned in the methods for FRNT_90_, 9 extracts were USUV-seropositive; 4 were flavivirus-seropositive; 1 was WNV-seropositive; and 41 were seronegative. Of the 9 USUV FRNT_90_-seropositive extracts, 5 were also USUV LMIA-reactive. Of the 4 flavivirus FRNT_90_-seropositive extracts, 1 was USUV and WNV LMIA-reactive and 3 were LMIA-non-reactive. The one WNV FRNT_90_-seropositive extract corresponds to the LMIA outlier that was excluded from further analysis. Of the 41 FRNT_90_-seronegative extracts, 10 were USUV LMIA-reactive (Supplementary Table S4).

Based on the classifications mentioned in the methods for IgY PMA, 4 extracts were USUV PMA-reactive; 4 were both USUV and WNV PMA-reactive; and 13 were PMA-non-reactive. Of the 4 USUV PMA-reactive extracts, 2 were also USUV LMIA-reactive. Of the 4 USUV and WNV PMA-reactive extracts, 2 were USUV LMIA-reactive, 1 was USUV and WNV LMIA-reactive and 1 was LMIA-non-reactive. Of the 13 PMA-non-reactive extracts, 6 were USUV LMIA-reactive (Supplementary Table S4).

54 USUV LMIA results were confirmed by USUV and WNV FRNT_90_ and a sub-set of 21 were also confirmed by USUV NS1 IgY PMA. Based on FRNT_90_ and PMA results, the LMIA correctly identified 46% (6/13) as seropositive and 63% (5/8) as reactive, respectively. Based on FRNT_90_ and PMA results, the LMIA correctly identified 76% (31/41) as seronegative and 54% (6/13) as non-reactive, respectively (Table 3). The LMIA did not have a statistically significant correlation with either the FRNT_90_ or PMA results (*p* = 0.1703 and 0.0850, respectively; Fisher’s exact test).

**Table 3:** A comparison of USUV NS1 LMIA results to FRNT_90_ and to USUV NS1 IgY PMA results.

		FRNT_90_		USUV NS1 IgY PMA	
		Pos.	Neg.	Reactive	Non-reactive
USUV NS1 LMIA	Reactive	6	10	5	6
	Non-reactive	7	31	3	7
Sensitivity		46%		63%	
Specificity		76%		54%	

## 4 Discussion

This study provides proof-of-concept evidence that an NS1-based LMIA can detect USUV NS1-specific antibodies in post-mortem tissues from naturally infected blackbirds found in the Netherlands. Blood clots and tissue extracts from lung and liver were analysed to evaluate the feasibility of antibody detection in these matrices, assess cross-reactivity with WNV NS1, and compare LMIA performance with established serological methods.

A substantial proportion of USUV RT-PCR–positive specimens did not test USUV LMIA-reactive or USUV FRNT_90_-seropositive. A likely explanation is that some birds experienced acute infection and died before mounting a detectable humoral response (Agliani et al., 2025; Münger et al., 2025). Conversely, one lung sample was RT-PCR–negative but USUV LMIA-reactive, illustrating that serological methods may detect evidence of prior infection when viral RNA is no longer present. Although based on a single sample, this finding supports the rationale for complementing molecular surveillance of deceased blackbirds with serology to reduce potential underreporting when viral clearance has occurred.

Among specimens for which multiple tissues were available, LMIA-reactivity was more frequently observed in lung extracts than in matched liver extracts. Given that USUV antigen has been reported across multiple organs in naturally infected blackbirds (Giglia et al., 2021) a smaller discrepancy between tissues might have been expected. The observed difference may reflect a combination of biological and technical factors. Lung tissue may retain higher residual blood content, resulting in greater antibody availability, whereas liver tissue contains higher levels of degradative enzymes and is subject to USUV-associated hepatic necrosis and inflammation (Agliani et al., 2023), which may accelerate post-mortem protein degradation. In addition, matrix effects may influence lateral flow performance, as differences in viscosity, protein composition, and debris can affect flow dynamics, background signal, and non-specific binding. In the present study, no visible background LMIA-reactivity was observed in FRNT_90_-confirmed seronegative tissue samples (Supplementary Figure S5), suggesting that non-specific binding was limited under the conditions used. However, the relative contributions of antibody abundance and matrix interference could not be distinguished. Future studies incorporating paired tissue samples, controlled infection models, and quantification of total immunoglobulin recovery would help clarify these effects and determine whether observed differences are driven by extraction efficiency or tissue-specific antibody distribution.

The rotational incubation protocol used in this study was adapted from previously described methods for antibody detection in lung tissue fragments (Sikkema et al., 2022) and was selected to preserve a simple, low-manipulation workflow. While more intensive homogenization or extended extraction procedures could increase antibody yield, particularly in denser tissues such as liver, such approaches would increase handling complexity and may generate aerosols when processing carcasses that could contain infectious virus. The selected protocol therefore represents a practical compromise between diagnostic yield, operator safety, and feasibility. Nevertheless, sample preprocessing remains a key determinant of assay performance, and variability in tissue sampling location, tissue mass, buffer-to-sample ratio, and extraction efficiency may contribute to differences observed between samples. Further optimization and standardization of preprocessing protocols will be important for improving comparability and sensitivity. While minimally invasive sampling strategies for live and deceased birds have been described (Atama et al., 2022), the relatively low volume of whole blood obtainable from feather tips would likely reduce LMIA diagnostic performance. Alternative field-compatible approaches, such as collecting blood swabs from within or near the lung cavity, may offer a more favourable balance between feasibility and diagnostic yield.

Serological differentiation between USUV and WNV remains challenging due to the high degree of amino acid homology between their NS1 and envelope proteins, and their co-circulation in parts of Europe (Calisher et al., 1989; Nikolay, 2015). In this study, the LMIA showed minimal cross-reactivity with WNV NS1 in tissue extracts, consistent with previous findings where the assay was able to distinguish between USUV NS1- and WNV NS1-specific antibodies (Godarzi et al., 2026, 2024). However, specificity assessment was limited to WNV NS1 antigen and to the available sample set which was comprised only of USUV-confirmed samples. Broader evaluation against additional flaviviruses relevant to the Netherlands such as tick-borne encephalitis virus (Esser et al., 2022) and a wider sample panel will be required to more comprehensively define assay specificity.

When compared with FRNT_90_, the LMIA showed limited concordance. This likely reflects fundamental differences in the antibody populations detected by the assays. Neutralization tests primarily measure functional antibodies targeting viral envelope proteins, whereas the LMIA detects binding antibodies against NS1, leading to potential discrepancies between NS1-based assays and neutralization assays (Atama et al., 2022; Münger et al., 2025; Streng et al., 2024). Several samples that were FRNT_90_-negative but LMIA-reactive were observed, suggesting that the LMIA may detect non-neutralizing or low-abundance antibodies not captured by neutralization assays.

In contrast to FRNT_90_, comparison between two NS1-based serological assays, LMIA and PMA, showed broadly similar performance, with slightly higher sensitivity but lower specificity for the LMIA. The reduced specificity may reflect the LMIA’s immunoglobulin class–independent design, in contrast to the IgY-specific PMA. Differences in assay format and incubation time may also contribute, as longer incubation in PMA may allow detection of antibodies with slower binding kinetics.

Reproducibility across duplicate capture spots was generally good, with some increased variability observed for weakly positive samples near the lower end of the assay range. Importantly, this did not alter the qualitative interpretation of the samples, indicating that the LMIA performs robustly for clear positives.

Several limitations should be considered. Sample sizes were small and uneven across tissue types, the interval between infection and death was unknown, and immunoglobulin recovery was not quantified. In addition, only blackbirds from the Netherlands were included, and no confirmed WNV-seropositive specimens were available for comparison. Post-mortem delay and environmental conditions may also influence antibody stability. Previous studies have similarly shown lower antibody titres in tissue extracts than in serum (Sikkema et al., 2022; Tryland et al., 2006; Waap et al., 2016), highlighting that tissue matrix effects should be considered when interpreting serological results from carcass-derived samples.

From an application perspective, the LMIA is a rapid, low-cost screening tool that complements established laboratory-based assays (Godarzi et al., 2026). Its double-antigen design (Supplementary Figure S1) enables species- and immunoglobulin class–independent detection, making it suitable for broad surveillance and for prioritizing samples for follow-up testing. Although the current workflow is laboratory-based, the proof-of-concept nature of this study supports further development toward field-compatible sampling strategies. For example, swabbing the lung cavity of carcasses and eluting the swab in assay buffer could reduce processing requirements and improve feasibility in field settings.

In conclusion, this study shows that USUV NS1-specific antibodies can be detected in post-mortem tissue extracts from blackbirds using an NS1-based LMIA. These findings establish carcass-derived tissue as a viable matrix for serological analysis and provide a foundation for further development of carcass-based surveillance strategies.

## Funding

This publication is part of the project ‘Preparing for Vector-Borne Virus Outbreaks in a Changing World: A One Health Approach’ (NWA.1160.1S.210), which is (partly) financed by the Dutch Research Council (NWO)).

## Declaration of generative AI use

During the preparation of this manuscript, the author (BG) used Perplexity for the sole purpose of improving language, clarity, and readability. After using this tool, the author carefully reviewed, edited, and adapted the text to ensure that the final submission represents their own analysis, interpretation, and original contribution. The author takes full responsibility for the content of the published article.

## CRediT authorship contribution statement

**Bijan Godarzi:** Writing – review & editing, Writing – original draft, Visualization, Validation, Software, Methodology, Investigation, Formal analysis, Conceptualization. **Cora M. Holicki:** Writing – review & editing, Investigation. **Felicity D. Chandler:** Writing – review & editing, Investigation. **Anne van der Linden:** Writing – review & editing, Investigation. **Reina S. Sikkema:** Writing – review & editing, Resources. **Edwin J.A. Veldhuizen:** Writing – review & editing, Supervision. **Aart van Amerongen:** Writing – review & editing, Supervision, Resources. **Andrea Gröne:** Writing – review & editing, Supervision, Resources.

## Declaration of competing interest

The authors declare that they have no known competing financial interests or personal relationships that could have appeared to influence the work reported in this paper.

## Data Availability

Data will be made available on request.

## Appendix Supplementary materials

Image, application 1
