# Supplementary material for: A lateral flow microarray immunoassay to detect USUV NS1-specific antibodies in tissues of deceased blackbirds in the Netherlands
Source: Virus Res. 2026 Aug 16;372:199789. doi: 10.1016/j.virusres.2026.199789 (PMC13524512; doi:10.1016/j.virusres.2026.199789)
Supplement: Supplementary file 1 [file mmc1.zip › Supplementary Figure S1.docx]

**Supplementary Figure S1** Schematic representations of A) tissue sample preparation as outlined in Methods section 2.3, B) LMIA assembly and microarray layout, and C) double antigen approach sandwiching an USUV NS1-specific antibody between two USUV NS1 antigens at the test spot and an anti-DNP antibody capturing a BSA-DNP conjugated CNP at the control spot.
