# Supplementary material for: A lateral flow microarray immunoassay to detect USUV NS1-specific antibodies in tissues of deceased blackbirds in the Netherlands
Source: Virus Res. 2026 Aug 16;372:199789. doi: 10.1016/j.virusres.2026.199789 (PMC13524512; doi:10.1016/j.virusres.2026.199789)
Supplement: Supplementary file 1 [file mmc1.zip › Supplementary Figure S3.docx]

**Supplementary Figure S3** Tissue sample selection workflow.
