# Supplementary material for: A lateral flow microarray immunoassay to detect USUV NS1-specific antibodies in tissues of deceased blackbirds in the Netherlands
Source: Virus Res. 2026 Aug 16;372:199789. doi: 10.1016/j.virusres.2026.199789 (PMC13524512; doi:10.1016/j.virusres.2026.199789)
Supplement: Supplementary file 1 [file mmc1.zip › Supplementary Figure S5.docx]

| Lung | Liver | Blood |
| --- | --- | --- |
|  |  |  |

**Supplementary Figure S5** LMIA-non-reactive samples do not show visible background spot development. The layout of the LMIA is shown in Supplementary Figure S1. All LMIA-non-reactive samples shown here are also confirmed seronegative and non-reactive in FRNT_90_ and PMA, respectively.
