# Supplementary material for: A lateral flow microarray immunoassay to detect USUV NS1-specific antibodies in tissues of deceased blackbirds in the Netherlands
Source: Virus Res. 2026 Aug 16;372:199789. doi: 10.1016/j.virusres.2026.199789 (PMC13524512; doi:10.1016/j.virusres.2026.199789)
Supplement: Supplementary file 1 [file mmc1.zip › Supplementary Table S4.docx]

**Supplementary Table S4** Complete table summarizing Date, Location, RT-PCR, LMIA, IgY PMA, and FRNT_90_ data.

| **ID** | **USUV**  **RT-PCR** | **Date**  **(YYYY-MM)** | **Province** | **Tissue** | **LMIA** | | **IgY PMA** | | **FRNT90** | | | |
| --- | --- | --- | --- | --- | --- | --- | --- | --- | --- | --- | --- | --- |
|  |  |  |  |  | **WNV NS1 (%CV)** | **USUV NS1 (%CV)** | **WNV NS1**  **(cut-off ≥ 20)** | **USUV NS1**  **(cut-off ≥ 20)** | **WNV**  **(cut-off ≥ 80)** | **USUV**  **(cut-off ≥ 160)** | **Result** |  |
| 1 | + | 2022-08 | Noord-Brabant | Lung | 0 | 0 | <20 | <20 | 10 | 10 | N |  |
|  |  |  |  | Liver | 0 | 0 | - | - | 10 | 20 | N |  |
|  |  |  |  | Blood | 0 | 0 | <20 | <20 | 10 | 10 | N |  |
| 2 |  | 2022-09 | Gelderland | Lung | 0 | 0 | <20 | <20 | 10 | 10 | N |  |
|  |  |  |  | Liver | 0 | 0 | - | - | 20 | 40 | N |  |
|  |  |  |  | Blood | 0 | 0 | <20 | <20 | 10 | 10 | N |  |
| 3 |  | 2022-08 | Flevoland | Lung | 0 | 0 | <20 | 109 | 10 | 20 | N |  |
|  |  |  |  | Liver | 0 | 0 | - | - | 10 | 10 | N |  |
|  |  |  |  | Blood | 0 | 1.21 (6%) | <20 | 66 | 10 | 10 | N |  |
| 4 |  | 2022-09 | Overijssel | Lung | 0 | 0.01 (141%) | 43 | 258 | 20 | 160 | USUV |  |
|  |  |  |  | Liver | 0 | 0 | - | - | 20 | 80 | N |  |
|  |  |  |  | Blood | 0 | 0.96 (19%) | 30 | 239 | 80 | 640 | USUV |  |
| 5 | + | 2020-08 | Gelderland | Lung | 0 | 0 | <20 | <20 | 10 | 20 | N |  |
|  |  |  |  | Liver | 0 | 0 | - | - | 10 | 10 | N |  |
| 6 |  | 2020-08 | Gelderland | Lung | 0 | 0.01 (77%) | <20 | <20 | 20 | 40 | N |  |
|  |  |  |  | Liver | 0 | 0.48 (7%) | - | - | 10 | 40 | N |  |
| 7 |  | 2020-08 | Gelderland | Lung | 0 | 0.35 (4%) | <20 | <20 | 10 | 20 | N |  |
|  |  |  |  | Liver | 0 | 0 | - | - | 40 | 80 | N |  |
| 8 |  | 2020-09 | Utrecht | Lung | 0 | 0.05 (94%) | <20 | <20 | 10 | 10 | N |  |
|  |  |  |  | Liver | 0 | 0 | - | - | 10 | 10 | N |  |
| 9 |  | 2021-08 | Utrecht | Lung | 0 | 0.11 (83%) | <20 | <20 | 20 | 40 | N |  |
|  |  |  |  | Liver | 0 | 0 | - | - | 10 | 20 | N |  |
| 10 |  | 2021-09 | Limburg | Lung | 0 | 1.62 (8%) | <20 | 481 | 80 | 320 | USUV |  |
|  |  |  |  | Liver | 0 | 0.18 (26%) | - | - | 40 | 160 | USUV |  |
| 11 |  | 2022-08 | Utrecht | Lung | 0 | 0 | <20 | <20 | 80 | 80 | FLAVI |  |
|  |  |  |  | Liver | 0 | 0 | - | - | 20 | 40 | N |  |
| 12 |  | 2022-09 | Noord-Brabant | Lung | 0 | 0.08 (7%) | <20 | <20 | 40 | 80 | N |  |
|  |  |  |  | Liver | 0 | 0.03 (141%) | - | - | 10 | 40 | N |  |
| 13 |  | 2022-09 | Zuid-Holland | Lung | 0 | 0.15 (87%) | <20 | <20 | 20 | 80 | N |  |
|  |  |  |  | Liver | 0 | 0 | - | - | 10 | 40 | N |  |
| 14 |  | 2022-08 | Noord-Brabant | Lung | 0 | 0 | <20 | 81 | 40 | 80 | N |  |
|  |  |  |  | Liver | 0 | 0 | - | - | 40 | 160 | USUV |  |
| 15 |  | 2022-09 | Noord-Brabant | Lung | 0 | 0 | <20 | <20 | 40 | 160 | USUV |  |
|  |  |  |  | Liver | 0 | 1.09 (11%) | - | - | 20 | 40 | N |  |
| 16 |  | 2022-08 | Gelderland | Lung | 0.33 (40%) | 1.34 (7%) | 55 | 66 | 80 | 80 | FLAVI |  |
|  |  |  |  | Liver | 0 | 0 | - | - | 80 | 160 | FLAVI |  |
| 17 |  | 2022-08 | Noord-Brabant | Lung | 0 | 0 | 30 | 239 | 40 | 80 | N |  |
|  |  |  |  | Liver | 0 | 0 | - | - | 20 | 80 | N |  |
| 18 |  | 2022-08 | Limburg | Lung | 0 | 0 | - | - | - | - | - |  |
|  |  |  |  | Liver | 0 | 0 | - | - | 40 | 80 | N |  |
| 19 | + | 2020-10 | Limburg | Blood | 0 | 0 | - | - | 10 | 10 | N |  |
| 20 |  | 2020-09 | North Brabant | Blood | 0 | 0 | - | - | 10 | 10 | N |  |
| 21 |  | 2020-09 | Gelderland | Blood | 0 | 0 | - | - | 10 | 10 | N |  |
| 22 |  | 2020-09 | Zuid-Holland | Blood* | 0 | 8.58 (4%) | - | - | 160 | 10 | WNV |  |
| 23 |  | 2020-08 | Gelderland | Blood | 0 | 0.42 (7%) | - | - | - | - | - |  |
| 24 | - | 2022-12 | Zuid-Holland | Lung | 0 | 0 | - | - | 10 | 10 | N |  |
|  |  |  |  | Liver | 0 | 0 | - | - | 40 | 160 | USUV |  |
| 25 |  | 2022-06 | Groningen | Lung | 0 | 0 | - | - | 10 | 10 | N |  |
|  |  |  |  | Liver | 0 | 0 | - | - | 10 | 40 | N |  |
|  |  |  |  | Blood | 0 | 0 | - | - | 10 | 10 | N |  |
| 26 |  | 2022-05 | Gelderland | Lung | 0 | 0 | - | - | 10 | 80 | N |  |
|  |  |  |  | Liver | 0 | 0 | - | - | 80 | 160 | FLAVI |  |
| 27 |  | 2022-01 | Friesland | Lung | 0 | 0 | - | - | 10 | 40 | N |  |
|  |  |  |  | Liver | 0 | 0 | - | - | 160 | 640 | USUV |  |
|  |  |  |  | Blood | 0 | 0 | - | - | 10 | 10 | N |  |
| 28 |  | 2022-01 | Flevoland | Lung | 0 | 0.04 (141%) | - | - | 80 | 320 | USUV |  |
|  |  |  |  | Liver | 0 | 0 | - | - | 40 | 80 | N |  |

*Excluded from any data analysis. Classifications for FRNT_90_ results are stated in section 3.4. Abbreviations: USUV = USUV-seropositive; WNV = WNV-seropositive; N = Seronegative; %CV = Intra-assay coefficient of variation.
